# Does Human Milk Modulate Body Composition in Late Preterm Infants at Term-Corrected Age?

**DOI:** 10.3390/nu8100664

**Published:** 2016-10-23

**Authors:** Maria Lorella Giannì, Dario Consonni, Nadia Liotto, Paola Roggero, Laura Morlacchi, Pasqua Piemontese, Camilla Menis, Fabio Mosca

**Affiliations:** 1 Fondazione I.R.C.C.S. Ca Granda Ospedale Maggiore Policlinico, Neonatal Intensive Care Unit, Department of Clinical Science and Community Health, University of Milan, Via Commenda 12, 20122 Milano, Italy; nadia.liotto@unimi.it (N.L.); paola.roggero@unimi.it (P.R.); lally.morly@hotmail.it (L.M.); pasquina.piemontese@mangiagalli.it (P.P.); camilla.menis@studenti.unimi.it (C.M.); fabio.mosca@unimi.it (F.M.); 2 Fondazione IRCCS Ca’ Granda Ospedale Maggiore Policlinico, Epidemiology Unit, Via San Barnaba 8, 20122 Milan, Italy; dario.consonni@unimi.it

**Keywords:** human milk, late preterm infants, body composition

## Abstract

(1) Background: Late preterm infants account for the majority of preterm births and are at risk of altered body composition. Because body composition modulates later health outcomes and human milk is recommended as the normal method for infant feeding, we sought to investigate whether human milk feeding in early life can modulate body composition development in late preterm infants; (2) Methods: Neonatal, anthropometric and feeding data of 284 late preterm infants were collected. Body composition was evaluated at term-corrected age by air displacement plethysmography. The effect of human milk feeding on fat-free mass and fat mass content was evaluated using multiple linear regression analysis; (3) Results: Human milk was fed to 68% of the infants. According to multiple regression analysis, being fed any human milk at discharge and at  term-corrected and being fed exclusively human milk at term-corrected age were positively associated with fat-free mass content(β = −47.9, 95% confidence interval (CI) = −95.7; −0.18; *p =* 0.049; β = −89.6, 95% CI = −131.5; −47.7; *p* < 0.0001; β = −104.1, 95% CI = −151.4; −56.7, *p* < 0.0001); (4) Conclusion: Human milk feeding appears to be associated with fat-free mass deposition in late preterm infants. Healthcare professionals should direct efforts toward promoting and supporting breastfeeding in these vulnerable infants.

## 1. Introduction

Late preterm birth, defined as a birth that occurs between 34 0/7 and 36 6/7 weeks of gestation, accounts for the majority of all preterm births [1]. Late preterm infants show increased mortality and morbidity compared with full-term newborn infants [2]. It has been reported that the first months of the postnatal life of late preterm infants are characterized by rapid postnatal catch-up growth, and as a result, at term-corrected age, late preterm infants achieve a weight either comparable to or higher than full-term newborns [3,4]. Evidence indicates that early body composition development in these infants is accompanied by a major deposition of fat mass so that, at term-corrected age, increased adiposity irrespective of the percentile at birth has been found. Unlike very preterm infants, however, late preterm infants appear not to develop a fat-free mass deficit [4]. 

It has long been recognized that early life represents a critical time window in terms of metabolic programming [5]. Indeed, increased adiposity early on may contribute to negative health outcomes later [6], whereas fat-free mass accretion has been positively associated with faster brain processing [7]. Considering the key role played by body composition development in modulating later health outcomes [6], identification of the determinants of body composition may help in tailoring nutritional interventions in infancy. Mode of feeding in early life has been reported to affect body composition development [8]. Human milk is recommended as the normal and unequalled method for feeding both preterm and term infants [9].

While some authors have investigated the determinants of body composition, including human milk feeding, in very preterm infants at the time of hospital discharge [10,11], there is a paucity of data on early determinants of body composition in late preterm infants who are recognized to be undergoing a critical period of development [12]. Huang et al. [8] have conducted a systematic review and meta-analysis, including infants born before completion of the 37th week of gestational age, to investigate whether body composition at term-corrected age differs between breastfed and formula-fed infants. However, late preterm infants were relatively underrepresented in the investigation. The aim of the present study was to investigate whether human milk consumption in early life could modulate body composition development at term-corrected age in late preterm infants. 

## 2. Materials and Methods 

### 2.1. Design and Setting

We conducted an observational cohort study. Approval was obtained from the institutional review board of Fondazione Istituto di Ricovero e Cura a Carattere Scientifico Cà Granda Ospedale Maggiore Policlinico (code number 506_2015, date of approval: 22 May 2015) and written informed consent from the infants’ parents. 

### 2.2. Sample

All consecutive newborns admitted to the authors’ institution between July 2015 and May 2016 were screened for eligibility. The inclusion criteria were gestational age from 34 0/7 to 36 6/7, Caucasian parentage and clinical stability at term-corrected age. The exclusion criteria were presence of congenital disease; chromosomal abnormalities; cardiac, brain, renal, endocrine, gastrointestinal or infectious disease; respiratory distress syndrome, defined as the need for any respiratory support for longer than seven days; and pre-pregnancy maternal body mass index >30.

### 2.3. Nutritional Practices

Infants were fed on demand. Mothers were encouraged either to breastfeed their infant or express their milk according to their infant’s clinical condition. According to our internal nutritional procedure, human milk was not fortified [13]. When human milk was unavailable or insufficient, formula feeding was started. Infants born at 34 weeks gestational age and infants born small for gestational age (SGA) at 35–36 weeks gestational age were fed a post-discharge formula (range of protein content: 2–2.4 g/100 mL; range of energy content: 73–82 kcal/100 mL) up to the maximum corrected age of 40 weeks. Late preterm infants, born adequate for gestational age (AGA), at 35–36 weeks of gestational age, were fed a regular-term formula (range of protein content: 1.3–1.7 g/100 mL; range of energy content: 66–68 kcal/100 mL). 

### 2.4. Data Collection 

Infants were enrolled at birth. At enrolment, basic subject characteristics such as gestational age at birth, anthropometrics parameters at birth and at discharge (weight, length and head circumference), gender, being a twin, and being adequate for gestational age or small for gestational age were recorded prospectively. Gestational age was based on the last menstrual period and first trimester ultrasonogram. Term-corrected age was calculated from the chronologic age, that is the time elapsed after birth, reduced by the number of weeks the infant was born before the expected date of delivery, that is, 40 weeks of gestation [14]. Infants with birth weight in the <10th or ≥10th percentile for gestational age, based on Fenton’s growth chart [15], were, respectively, classified as having weight that was SGA or AGA. The feeding status at discharge (any human milk, including exclusively human milk or exclusively formula) and length of hospital stay were also collected. Specifically, infants fed any extent of human milk, irrespective of the quantity or the exclusivity, were categorized as fed any human milk [9]. After discharge, the parents were asked to report in a diary the mode of feeding from discharge up to term-corrected age (40 weeks ± 2 days). 

### 2.5. Growth and Body Composition Assessment

Anthropometric measurements were assessed at birth, at discharge and at term-corrected age (40 weeks ± 2 days). Body weight, length and head circumference were measured according to standard procedures [16]. The weight of each baby was measured on an electronic scale accurate to 0.1 g (PEA POD Infant Body Composition System; Cosmed, Concord, CA, USA). Body length was measured to the nearest 1 mm on a Harpenden neonatometer (Holtain, Crymych, UK). Head circumference was measured to the nearest 1 mm using non-stretch measuring tape. All measurements were assessed by trained medical staff of the author’s institution. The late preterm infants’ growth (weight, length and head circumference) *z*-scores were then calculated using the *z*-score calculator provided by the University of Calgary, Calgary, Alberta, Canada [17]. Body composition was assessed at term-corrected age using an air-displacement plethysmograph (PEA POD Infant Body Composition System; COSMED, Concord, CA, USA). A detailed description of the PEA POD’s physical design, operating principles, validation, and measurement procedures is provided elsewhere [18,19]. Briefly, the PEA POD assesses fat mass and fat-free mass by direct measurements of body mass and volume and the application of a classic densitometric model where percentage of body fat is calculated using body density and pre-determined fat and fat-free mass density values. Body fat was defined as body weight minus fat-free mass. A constant fat mass density value of 0.9007 g/mL [20,21] is used. Fat-free mass density values are calculated as the sum of the contribution of the various components in the fat-free mass compartment. Age- and sex-specific fat-free mass density values extrapolated from data by Fomon et al. are used [22].

### 2.6. Statistical Analysis

All descriptive data are expressed as the mean ± SD or *n* (%). The associations between neonatal characteristics (anthropometric measurements at birth and at term-corrected age, feeding status at discharge and at term-corrected age), fat mass and fat-free mass content at term-corrected age were assessed using univariate linear regression analysis. Multiple linear regression models, including variables that resulted to be significant at univariate analysis, were used to identify the determinants of fat mass and fat free mass content at term-corrected age. In order to avoid collinearity, with regard to anthropometric parameters, we included only weight at term-corrected age as independent variable since it was most closely correlated with fat-free mass and fat mass content at term-corrected age in the univariate analysis. Weight was expressed as *z*-scores, in order to take into account gestational age. All statistical analyses were performed using SPSS (SPSS, version 12; SPSS, Chicago, IL, USA) and Stata (StataCorp. 2013, Stata Statistical Software: Release 13. StataCorp LP, College Station, TX, USA).

## 3. Results

**Figure 1:**
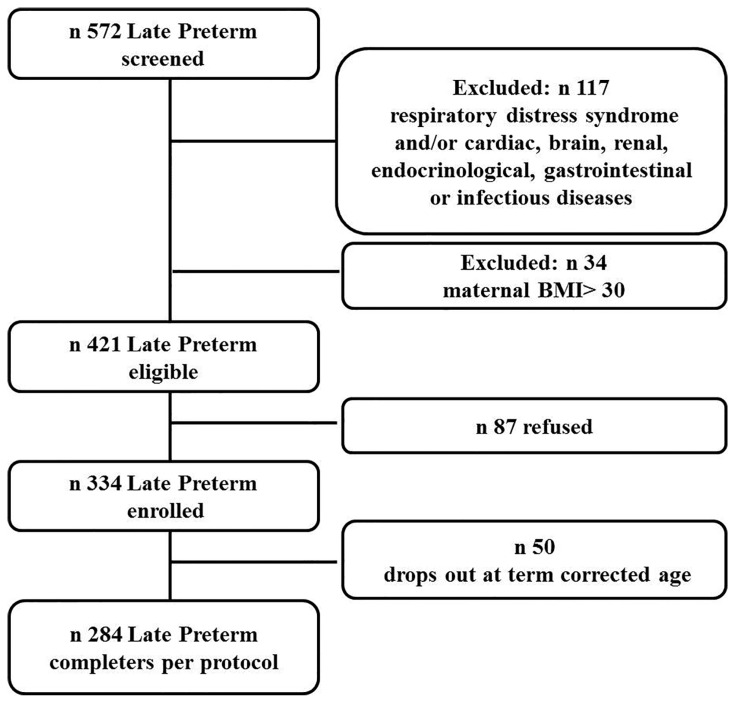
Flow chart of the study.

A total of 284 late preterm infants were enrolled. The flow chart of the study is reported in Figure 1. The mean hospital stay was 8.9 ± 5.05 days. The mean postmenstrual age at discharge was 36.6 ± 0.8 weeks. The mean chronological age at term-corrected age was 33.1 ± 3.6 days.

The basic characteristics of the subjects at birth are shown in Table 1. 

Mode of feeding, anthropometric parameters and body composition in the enrolled late preterm infants at discharge and at term-corrected age are reported in Table 2 and Table 3, respectively.

In the univariate analysis, anthropometric parameters at birth and at term-corrected age, gestational age, being male and being fed human milk at discharge and at term-corrected age were all positively associated with fat-free mass content at term-corrected age, whereas being born small for gestational age and being a twin were negatively associated. With regard to fat mass, anthropometric parameters at birth and at term-corrected age and being exclusively fed human milk at discharge were positively associated with fat mass content at term-corrected age, whereas being born small for gestational age was negatively associated (Table 4). 

At multiple regression analysis, when including mode of feeding at discharge, being male, weight *z*-score at term-corrected age and being fed any human milk at discharge were positively associated with fat-free mass content at term-corrected age (Table 5 and Table 6). 

With regard to fat mass content, the weight *z*-score at term-corrected age was positively associated with its content at term-corrected age, whereas the mode of feeding was not significantly associated (Table 7).

In the multiple regression analysis, when including the mode of feeding at term-corrected age, being male, the weight *z*-score at term-corrected age and being fed either exclusively or any human milk at term-corrected age were positively associated with fat-free mass content at term-corrected age (Table 8 and Table 9).

## 4. Discussion

The findings of this study indicate that the consumption of human milk is associated with fat-free mass deposition in late preterm infants. It must be taken into account that the strength of this relationship appears to become stronger towards the achievement of term-corrected age, suggesting a potential cumulative effect of human milk consumption on body composition development. 

Fat-free mass content has been recognized to positively modulate central nervous system development because greater fat-free mass gains during the hospital stay have been associated with improved cognitive and motor scores at one year of corrected age in very-low-birth-weight infants [23]. In addition, higher fat-free mass content in former preterm infants at four months of corrected age was found to be associated with shorter brain speed processing [7]. On the other hand, the consumption of human milk at term-corrected age was found to be negatively associated with fat mass deposition. Considering that late preterm infants are at risk for developing increased adiposity at term-corrected age [3,4], which in turn could represent a risk factor for developing metabolic syndrome later in life, it could be speculated that human milk exerts a potentially protective effect in late preterm infants against obesity risk. 

The present results are consistent with previous data published in the literature. Larcade et al. [11] reported that the number of days very preterm infants were fed human milk during their hospital stay positively correlated with fat-free mass content at discharge. Accordingly, Huang et al. [8] conducted a meta-analysis investigating the effect of breastfeeding and formula feeding on the body composition of 642 preterm infants (<37 weeks of gestational age). The authors found significantly lower fat mass in breastfed infants in comparison to formula-fed infants (mean difference 0.24; 95% CI 0.17, 0.31 kg) at term-corrected age. These results differ from those reported by Gale et al. [24] who found significantly higher fat mass content in healthy, full-term, breastfed infants at three to four months of age in comparison to formula-fed infants. However, it must be taken into account that preterm infants, including those born between 34 and 36 weeks of gestation, still need to complete their organ development and present a lack of both fat-free mass and fat mass at birth because premature birth interrupts the physiologic development of body composition [25]. Furthermore, it has been demonstrated that the early postnatal growth of late preterm infants is characterized by a major deposition of fat mass so that at term-corrected age, late preterm infants show a higher fat mass content than term infants [3,4]. Hence, the promotion of fat-free mass accretion by consumption of human milk may represent a physiological compensatory mechanism aimed at recovering body composition, promoting neurodevelopment and protecting preterm infants from potential unbalanced nutritional intake provided by infant formulas. 

In the present study, not surprisingly, weight *z*-scores at birth and at term-corrected age were positively associated with fat-free mass at term-corrected age. Indeed, the fat-free mass compartment accounts for the majority of the weight [25]. In addition, the finding that weight *z*-scores at term-corrected age are positively associated with fat mass content at term-corrected age can be explained by the fact that the early postnatal growth of late preterm infants is characterized by a major deposition of fat mass [4]. Consistently with previous data reported in the literature [26], being male was positively associated with fat-free mass content at term-corrected age. 

While this study is of clinical interest, it presents some limitations. First of all, the data collected in the present study refer to late preterm infants not affected by comorbidities, so that their effect on body composition development has not been assessed. Furthermore, body composition was evaluated only at one time point, and as a result, the effect of human milk feeding over time has not been investigated. The main strength of the paper is represented by the fact that body composition data have been collected from a large number of late preterm infants. 

## 5. Conclusions 

The findings from this study indicate that human milk feeding appears to be associated with early fat-free mass deposition in late preterm infants. On the basis of the present results, health care professionals should direct efforts toward promoting and supporting breastfeeding in these vulnerable infants. Future research is desirable to explore the effect of comorbidities and the persistence of the effect of human milk consumption on body composition development in late preterm infants.

## Figures and Tables

**Table 1:** Basic subject characteristics at birth.

	Late Preterm Infants (*n* = 284)	Males (*n* = 147)	Females (*n* = 137)
Gestational age (weeks)	35.3 ± 0.8	35.2 ± 0.9	35.4 ± 0.7
Birth weight (g)	2413 ± 387	2427 ± 404	2397 ± 369
Length (cm)	45.3 ± 2.4	45.3 ± 2.6	45.3 ± 2.2
Head circumference (cm)	31.9 ± 1.4	31.9 ± 1.6	31.9 ± 1.2
Weight *z*-scores	−0.23 ± 0.87	−0.27 ± 0.8	−0.28 ± 0.8
Length *z*-scores	−0.33 ± 0.91	−0.31 ± 0.9	−0.28 ± 0.8
Head circumference *z*-scores	−0.09 ± 0.88	−0.06 ± 0.9	−0.01 ± 0.8
Small for gestational age *n* (%)	42 (15)	18 (12)	24 (18)
Twins *n* (%)	119 (42)	50 (34)	69 (50)

Data are presented as the mean (SD) or *n* (%).

**Table 2:** Mode of feeding and anthropometric parameters of the enrolled late preterm infants at discharge.

	Late Preterm Infants (*n* = 284)	Males (*n* = 147)	Females (*n* = 137)
Exclusive human milk *n* (%)	97 (34)	53 (36)	44 (32)
Exclusive formula *n* (%)	91 (32)	35 (24)	56 (41)
Any human milk *n* (%)	193 (68)	112 (77)	81 (59)
Weight (g)	2270 ± 497	2317 ± 442	2200 ± 428
Length (cm)	45.1 ± 2.7	45.1 ± 3.4	45.1 ± 2.2
Head circumference (cm)	31.9 ± 1.4	31.9 ± 1.5	31.8 ± 1.1
Weight *z*-scores	−0.92 ± 0.8	−0.95 ± 0.8	−0.94 ± 0.8
Length *z*-scores	−0.70 ± 0.9	−0.85 ± 1.0	−0.53 ± 0.8
Head circumference *z*-scores	−0.38 ± 0.7	−0.51 ± 0.8	−0.30 ± 0.7

**Table 3:** Mode of feeding, anthropometric parameters and body composition of the enrolled late preterm infants at term-corrected age.

	Late Preterm Infants (*n* = 284)	Males (*n* = 147)	Females (*n* = 137)
Exclusive human milk *n* (%)	88 (31)	54 (37)	34 (25)
Exclusive formula *n* (%)	134 (47)	68 (46)	66 (48)
Any human milk *n* (%)	150 (53)	79 (54)	71 (52)
Weight (g)	3396 ± 504	3380 ± 526	3240 ± 521
Length (cm)	49.4 ± 2.3	49.7 ± 2.5	49.1 ± 2.2
Head circumference (cm)	35.1 ± 1.6	35.3 ± 2.0	34.7 ± 1.3
Weight *z*-scores	−0.31 ± 1.1	−0.38 ± 1.1	−0.23 ± 1.0
Length *z*-scores	−0.56 ± 1.0	−0.59 ± 1.1	−0.53 ± 0.9
Head circumference *z*-scores	0.14 ± 0.9	0.25 ± 0.9	−0.02 ± 0.9
Fat mass %	14.7 ± 4.7	14.2 ± 4.5	15.3 ± 4.9
Fat free mass %	85.2 ± 4.8	85.8 ± 4.6	84.7 ± 4.9
Fat mass (g)	510 ± 2.1	493.8 ± 204	527.6 ± 219
Fat free mass (g)	2878 ± 392	2934.2 ± 408	2817.1 ± 366

**Table 4:** Univariate linear regression analysis for associations of infant characteristics, anthropometric parameters and mode of feeding with fat-free mass and fat mass at term-corrected age.

Parameters	Fat-Free Mass at Term-Corrected Age (g)			Fat Mass at Term-Corrected Age (g)		
	β	95% Confidence Interval	*p*	β	95% Confidence Interval	*p*
Gestational age (weeks)	64.5	8.1; 120.9	0.025	−5.7	−36.5; 25.0	0.713
Being male (yes vs. no)	117.0	26.1; 207.8	0.012	−33.8	−83.2; 15.7	0.180
Being small for gestational age (no vs. yes)	−401.9	−522.3; −281.5	<0.0001	−128.5	−196.8; 60.3	<0.0001
Weight *z*-score at birth	280.9	238.1; 323.7	<0.0001	92.5	65.4; 119.6	<0.0001
Length *z*-score at birth	175.0	129; 220.9	<0.0001	60.8	34.5; 87.1	<0.0001
Head circumference *z*-score at birth	156.4	107.3; 205.4	<0.0001	57.5	29.9; 85.0	<0.0001
Weight *z*-score at term-corrected age	307.1	283.9; 330.2	<0.0001	140.3	124.1; 156.6	<0.0001
Length *z*-score at term-corrected age	246.4	212.4; 280.4	<0.0001	103.6	82.7; 124.4	<0.0001
Head circumference *z*-score at term-corrected age	232.5	191.3; 273.6	<0.0001	87.5	62.8; 112.1	<0.0001
Being twin (no vs. yes)	−187.6	−289.6; −85.6	<0.0001	−32.8	−89.8; 24.8	0.256
Being exclusively human milk fed vs. exclusively formula fed at discharge	−333	−483.6; −183.5	<0.0001	−99.1	−179.1; −19.1	0.016
Being fed any human milk vs. exclusively formula fed at discharge	−223	−337.9; −109.0	<0.0001	−43.6	−110.0; 22.9	0.197
Being exclusively human milk fed vs. exclusively formula fed at term-corrected age	−226	−344.6; 109.1	<0.0001	17.8	−48.1; 83.8	0.594
Being fed any human milk vs. exclusively formula fed at term-corrected age	−227	−327.8; −126.2	<0.0001	−7.8	−65.0; 49.4	0.788

**Table 5:** Multiple linear regression analysis for associations of gender, weight *z*-score at term-corrected age, being a twin and being exclusively fed human milk at discharge with fat-free mass at term-corrected age (*R*^2^ = 0.88, *p* < 0.0001).

Model	Fat-Free Mass Content at Term-Corrected Age (g)		
	β	95% Confidence Interval	*p*
Intercept	3008.4	2898.5; 3118.3	<0.0001
Male (yes vs. no)	80.6	20.6; 140.6	0.009
Being a twin (no vs. yes)	−7.9	−73.4; 57.6	0.8
Weight *z*-score at term-corrected age	361.2	327.9; 394.6	<0.0001
Mode of feeding at discharge (being exclusively human milk fed vs. being exclusively formula fed)	−39.6	−102.4; 23.2	0.214

**Table 6:** Multiple linear regression analysis for associations of gender, weight *z*-score at term-corrected age, being a twin and being fed any human milk at discharge with fat-free mass at term-corrected age (*R*^2^ = 0.85, *p* < 0.0001).

Model	Fat-Free Mass Content at Term-Corrected Age (g)		
	β	95% Confidence Interval	*p*
Intercept	3037.2	2961.2; 3113.1	<0.0001
Male (yes vs. no)	68.0	24.2; 111.7	0.002
Being a twin (no vs. yes)	−38.7	−82.3; 4.7	0.08
Weight *z*-score at term-corrected age	347.7	325.2; 370.2	<0.0001
Mode of feeding at discharge (being fed any human milk vs. being exclusively formula fed)	−47.9	−95.7; −0.18	0.049

**Table 7:** Multiple linear regression analysis for associations of weight *z*-score at term-corrected age, and being exclusively human milk fed at discharge with fat mass at term-corrected age (*R*^2^ = 0.63, *p* < 0.0001).

Model	Fat Mass Content at Term-Corrected Age (g)		
	β	95% Confidence Interval	*p*
Intercept	517.7	430.8; 604.5	<0.0001
Weight *z*-score at term-corrected age	164.9	137.8; 192.0	<0.0001
Mode of feeding at discharge (being exclusively human milk fed vs. being exclusively formula fed)	27.2	−27.0; 81.5	0.321

**Table 8:** Multiple linear regression analysis for associations of gender, weight *z*-score at term-corrected age, being a twin and being fed exclusively human milk at term-corrected age with fat-free mass at term-corrected age (*R*^2^ = 0.88, *p* < 0.0001).

Model	Fat-Free Mass Content at Term-Corrected Age (g)		
	β	95% Confidence Interval	*p*
Intercept	3098.2	3018.1; 3178.3	<0.0001
Male (yes vs. no)	84.3	41.1; 127.4	<0.0001
Being a twin (no vs. yes)	23.4	−23.6; 70.4	0.32
Weight *z*-score at term-corrected age	355.0	332.8; 377.2	<0.0001
Mode of feeding at term-corrected age (being exclusively human milk fed vs. being exclusively formula fed)	−104.1	−151.4; −56.7	<0.0001

**Table 9:** Multiple linear regression analysis for associations of gender, weight *z*-score at term-corrected age, being a twin and being fed any human milk at term-corrected age with fat-free mass at term-corrected age (*R*^2^ = 0.86, *p* < 0.0001).

Model	Fat-Free Mass Content at Term-Corrected Age (g)		
	β	95% Confidence Interval	*p*
Intercept	3088.1	3023.3; 3152.9	<0.0001
Male (yes vs. no)	75.0	34.8; 115.2	<0.0001
Being a twin (no vs. yes)	1.5	−41.2; 44.2	0.94
Weight *z*-score at term-corrected age	354.5	333.7; 375.3	<0.0001
Mode of feeding at term-corrected age (being fed any human milk vs. being exclusively formula fed)	−89.6	−131.5; −47.7	<0.0001
